# The epidemiological and histopathological factors for delayed local recurrence in oral squamous cell carcinoma

**DOI:** 10.1186/s40902-024-00443-8

**Published:** 2024-11-12

**Authors:** Hyosik Kim, Sang-Min Lee, Kang-Min Ahn

**Affiliations:** Department of Oral and Maxillofacial Surgery, College of Medicine, Asan Medical Center, University of Ulsan, Seoul, South Korea

**Keywords:** Oral squamous cell carcinoma, Local recurrence, Histopathological factors, Histological grading, Recurrence intervals

## Abstract

**Background:**

Oral squamous cell carcinoma (OSCC) is a prevalent malignancy in oral cancer. Approximately 26% of OSCC cases recur after initial curative treatment, with over 80% of these recurrences occurring within the first 2 years. Delayed local recurrence (DLR) occurring beyond the 2-year period in cases of OSCC is infrequent. The aim of this study is to investigate the histopathological characteristics associated with DLR.

**Methods:**

This study included 197 patients diagnosed with OSCC who underwent primary surgery from 2006 to 2022. Epidemiological features, such as age and gender, and histopathological features, including primary tumor sites, TNM staging, histopathological grading, depth of invasion, the presence of lymphovascular or perineural invasion, and the utilization of radiation therapy (RT) and neck dissection (ND) were analyzed.

**Results:**

The mean interval until local recurrence was 22 ± 27 months. There were 10 cases of DLR (20.83%) among 51 patients with local recurrence. The epidemiological and histopathological analysis of these cases is as follows: 10 patients (3 males and 7 females) aged 51–80 years (median, 56.5 years). Primary tumor sites were tongue (*n* = 3), maxillary gingiva (*n* = 1), mandibular gingiva (*n* = 3), retromolar trigone (*n* = 1), and buccal mucosa (*n* = 2). Tumor size was advanced (T3/T4) in 5 cases, while a smaller size (T1/T2) was observed in 5 cases. No lymph node metastasis was 80.0%. Histopathological grading was well differentiated in 9 cases and moderately differentiated in 1 case, with no cases of poorly differentiated tumors. Depth of invasion > 5 mm was 70.0% of the cases (*n* = 7). Lymphovascular invasion and perineural invasion were not present. Three patients received RT, and 8 patients underwent ND. There were 2 patients who consumed alcohol, and 2 patients who smoked tobacco. The results showed that histological differentiation had a significant relationship with the interval (*p* = 0.031).

**Conclusions:**

DLR, occurring more than 2 years after the initial tumor resection surgery, is infrequent. Histological differentiation is associated with tumor recurrence intervals. Patients with a higher histological grading require more precise follow-up observation during the initial 2 years after surgery.

## Background

Oral cancer is the sixth most common cancer worldwide [1, 2]. Oral squamous cell carcinoma (OSCC) is a prevalent malignancy in oral cancer. Recurrence is an important prognostic factor in patients with OSCC. The presence of recurrence significantly impacts the 5-year relative survival rate. One study reported that OSCC patients without recurrence had a relative survival rate of 92%, whereas those with recurrence had only 30% [3]. Therefore, identifying factors that contribute to recurrence is crucial for improving patient outcomes. In previous studies, it was observed that approximately 26% of OSCC cases recur after initial curative treatment, with more than 80% of these recurrences occurring within the first 2 years [4, 5]. Thus, delayed local recurrences (DLR) occurring beyond the 2-year period in cases of OSCC are infrequent.

The aim of this study is to analyze the factors associated with DLR. We hypothesize that epidemiological and histopathological features are correlated with tumor recurrence intervals. This study is believed to provide assistance in understanding DLR and predicting the timing of recurrences, which can aid in formulating patient-specific surveillance plans.

## Methods

This study included 197 patients diagnosed with OSCC who underwent primary surgery performed by one surgeon from 2006 to 2022. This research was approved by the Institutional Review Board under the number S2023-2015–0001 at our institute. Patients with malignancies other than OSCC upon postoperative final histopathological examination were excluded from the study. Inoperable patients due to their general condition, tumor status, and/or distant metastases were assigned to chemotherapy or radiation therapy (RT) and were excluded from the study.

Recurrence was defined as follows: (1) local recurrence—recurrence at the same anatomic site after primary treatment, (2) regional recurrence—lymph node metastases of the neck after primary treatment, (3) distant metastases—metastases in the body distant or remote from the primary tumor, (4) second primary cancer of the oral cavity—malignancies at different anatomic sites within the oral cavity after primary treatment. In this study, cases of local recurrence more than 2 years after primary treatment were classified as DLR. In this study, combination refers to the simultaneous occurrence of two or more of the four types of recurrences.

We analyzed epidemiological features such as age, gender, risk factors like alcohol or tobacco, and histopathological features including primary tumor site, TNM classification, histopathological grade, depth of invasion, the presence of lymphovascular or perineural invasion, and the utilization of RT and neck dissection (ND). IBM SPSS Statistics 21 (IBM Corp., Armonk, NY, USA) was used for statistical analysis. The chi-squared test was used to assess the correlations between each variable and both ELR and DLR. Additionally, binary logistic regression analysis was employed to analyze factors influencing ELR and DLR. One-way ANOVA was conducted to identify potential factors associated with the recurrence interval. Finally, Kaplan–Meier plots, in conjunction with log-rank tests, were used to analyze the survival rates of patients who experienced local recurrence. Statistical significance was defined as *p*-values less than or equal to 0.05.

## Results

### General information

**Fig. 1:**
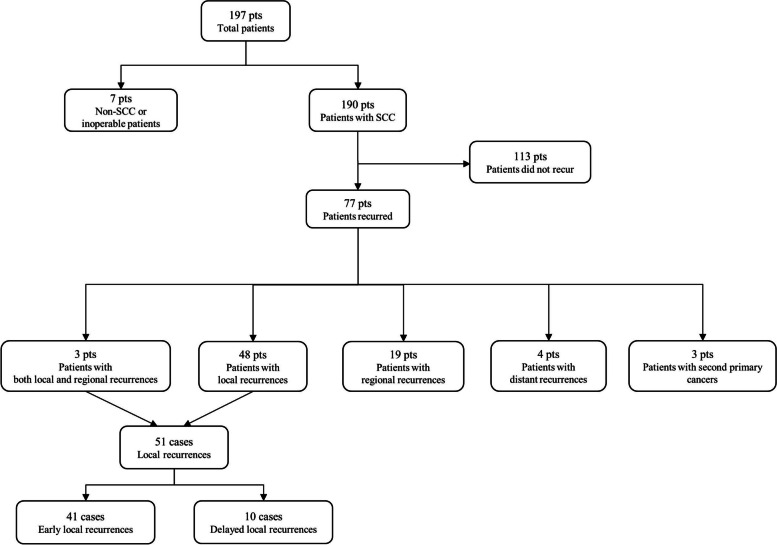
Flowchart of patients and recurrences included in the study

Among the total of 197 patients, 7 patients met the exclusion criteria due to histological evidence and inoperability. Out of the 190 patients diagnosed with OSCC, the overall recurrence rate, including all those who experienced any type of recurrence, was 40.52% (*n* = 77). The average interval until tumor recurrence was 17 ± 23 months. Within the subset of 77 recurrence patients, 48 patients (62.34%) experienced local recurrences, 19 patients (24.68%) had regional recurrences, 4 patients (5.19%) experienced distant recurrences, 3 patients (3.90%) were diagnosed with second primary cancers, and 3 patients (3.90%) presented with a combination of local and cervical lymph node metastases. We analyzed a total of 51 cases of local recurrence, including 48 cases of sole local recurrence and 3 cases of local recurrence with concurrent neck metastasis (Fig. 1). The mean interval until overall local recurrence was 20 ± 25 months. Among local recurrences, there were 17 cases (33.33%) of recurrence within 6 months, 28 cases (54.90%) within 12 months, and 41 cases (80.39%) within 2 years. We defined cases where recurrence occurred within 2 years after surgery as ELR and cases where recurrence happened beyond 2 years as DLR. There were 10 cases of DLR, accounting for 19.61% of the total local recurrences. The mean interval for DLR was 66 ± 25 months, while that for ELR was 10 ± 7 months. The epidemiological and histopathological analysis of the 10 patients with DLR of SCC is as follows: 10 patients (3 males and 7 females) aged 51–80 years (median, 56.5 years). Primary tumor sites were tongue (*n* = 3), maxillary gingiva (*n* = 1), mandibular gingiva (*n* = 3), retromolar trigone (RMT) (*n* = 1), and buccal mucosa (*n* = 2). Tumor size was advanced (T3/T4) in 5 cases, while a smaller primary tumor size (T1/T2) was observed in 5 cases. No lymph node metastasis (N0) was 80.0%. There were well-differentiated (G1) in 9 cases and moderately differentiated (G2) in 1 case, but there were no cases of poorly differentiated tumors (G3). Depth of invasion > 5 mm was 70.0% of the cases (*n* = 7). Lymphovascular and perineural invasion were not present. Three patients received RT, and 8 patients underwent ND. There were 2 patients who consumed alcohol, and 2 patients who smoked tobacco.

### Recurrence factors

According to the chi-squared test results, variables that showed a significant correlation with recurrence (regardless of the type of recurrence) were T stage (*p* = 0.003), N stage (*p* = 0.000), DOI (*p* = 0.002) and RT utilization (*p* = 0.001) (Table 1). The analysis of variables and their correlation with early recurrence and delayed recurrence among the 51 patients who experienced local recurrence did not yield statistically significant results (Table 2). A binary logistic regression was conducted to identify factors influencing early and delayed local recurrence, but no significant factors were found (Table 3). The results of the ANOVA analysis conducted to examine the correlation between variables and the interval to recurrence indicate that histological differentiation had a significant relationship with the interval (*p* = 0.031) (Table 4). Specifically, lower histological grading was associated with a longer time to local recurrence (Table 5).

**Table 1:** The chi-squared test in 190 patients with oral squamous cell carcinoma (OSCC)

Variable					*p* value
Age (year)		Cases	Recur	Non-recur	0.991
	≤ 60	69	28	41	
	> 60	121	49	72	
Gender		Cases	Recur	Non-recur	0.095
	Male	129	47	82	
	Female	61	30	31	
Primary tumor site		Cases	Recur	Non-recur	0.657
	Lip	2	1	1	
	Tongue	39	14	25	
	FOM	17	6	11	
	Mx. gingiva	26	11	15	
	Mn. gingiva	47	16	31	
	RMT	13	6	7	
	Palate	5	2	3	
	BM	30	13	17	
	PIOS	10	7	3	
	Unclassified	1	1	0	
T stage		Cases	Recur	Non-recur	0.002*
	1	39	8	31	
	2	51	17	34	
	3	20	13	7	
	4	80	39	41	
pN state		Cases	Recur	Non-recur	0.000*
	0	130	40	90	
	1	23	12	11	
	2	34	22	12	
	3	3	3	0	
Histological grade		Cases	Recur	Non-recur	0.927
	G1	92	36	56	
	G2	93	39	54	
	G3	5	2	3	
Depth of invasion (DOI)		Cases	Recur	Non-recur	0.002**
	≤ 5	80	22	58	
	> 5	110	55	55	
Lymphovascular invasion		Cases	Recur	Non-recur	0.949
	No	160	65	95	
	Yes	30	12	18	
Perineural invasion		Cases	Recur	Non-recur	0.362
	No	170	67	103	
	Yes	20	10	10	
Alcohol		Cases	Recur	Non-recur	0.630
	No	117	49	68	
	Yes	73	28	45	
Tobacco		Cases	Recur	Non-recur	0.082
	No	104	48	56	
	Yes	86	29	57	
Neck dissection		Cases	Recur	Non-recur	0.767
	No	69	27	42	
	Yes	121	50	71	
Radiation therapy		Cases	Recur	Non-recur	0.001*
	No	111	34	77	
	Yes	79	43	36	
	Total	190	77	113	

^*^Since the *p* value is less than 0.05, it is statistically significant

^**^When not divided by the DOI interval, the value is 0.057

**Table 2:** The chi-squared test in 51 patients with local recurrence (early vs. delayed)

Variable		*N* = 51	*N* = 41	*N* = 10	*p* value
Age (year)		Cases	Early	Delayed	0.097
	≤ 60	19	13	6	
	> 60	32	28	4	
Gender		Cases	Early	Delayed	0.597
	Female	33	26	7	
	Male	18	15	3	
Primary tumor site		Cases	Early	Delayed	0.856
	Lip	1	1	0	
	Tongue	11	8	3	
	FOM	2	2	0	
	Mx. gingiva	9	8	1	
	Mn. gingiva	11	8	3	
	RMT	3	2	1	
	BM	10	8	2	
	PIOS	4	4	0	
T stage		Cases	Early	Delayed	0.437
	1	4	3	1	
	2	12	8	4	
	3	4	4	0	
	4	31	26	5	
pN state		Cases	Early	Delayed	0.538
	0	33	25	8	
	1	5	5	0	
	2	11	9	2	
	3	2	2	0	
Histological grade		Cases	Early	Delayed	0.081
	G1	30	21	9	
	G2	19	18	1	
	G3	2	2	0	
Depth of invasion (DOI)		Cases	Early	Delayed	0.840
	≤ 5	14	11	3	
	> 5	37	30	7	
Lymphovascular invasion		Cases	Early	Delayed	0.304
	No	47	37	10	
	Yes	4	4	0	
Perineural invasion		Cases	Early	Delayed	0.304
	No	47	37	10	
	Yes	4	4	0	
Alcohol		Cases	Early	Delayed	0.387
	No	35	27	8	
	Yes	16	14	2	
Tobacco		Cases	Early	Delayed	0.208
	No	32	24	8	
	Yes	19	17	2	
Neck dissection		Cases	Early	Delayed	0.657
	No	12	10	2	
	Yes	39	31	8	
Radiation therapy		Cases	Early	Delayed	0.180
	No	25	18	7	
	Yes	26	23	3	
	Total	51	41	10

**Table 3:** Binary logistic regression for factors influencing local recurrence (early vs. delayed)

	Variable	
		*p* value Exp(B)
Local recurrence (early or delayed)	Age Gender	0.475 0.362 0.377 0.965
	Primary tumor site	0.399 0.807
	T stage	0.788 0.867
	pN state	0.838 0.896
	Histological grade	0.181 0.174
	Depth of invasion (DOI)	0.201 0.918
	Lymphovascular invasion	0.999 0.000
	Perineural invasion	0.999 0.000
	Alcohol	0.672 0.580
	Tobacco	0.400 0.292
	Neck dissection	0.370 2.947
	Radiation therapy	0.228 0.249

**Table 4:** One-way ANOVA analysis of variables and the interval to local recurrence

Variable	*p* value
Age	0.375
Gender	0.961
Primary tumor site	0.979
T stage	0.240
pN state	0.450
Histological grade	0.031
Depth of invasion (DOI)	0.342
Lymphovascular invasion	0.290
Perineural invasion	0.255
Alcohol	0.413
Tobacco	0.214
Neck dissection	0.634
Radiation therapy	0.262

**Table 5:** The mean interval to local recurrence based on histological differentiation

Interval * Histological grade			
Interval			
Grade	Mean (month)	*N*	Std. deviation
G1 (well differentiation)	29.63	30	31.545
G2 (moderately differentiation)	10.58	19	9.329
G3 (poorly differentiation)	6.00	2	2.828
Total	21.61	51	26.519

### Survival analysis

**Fig. 2:**
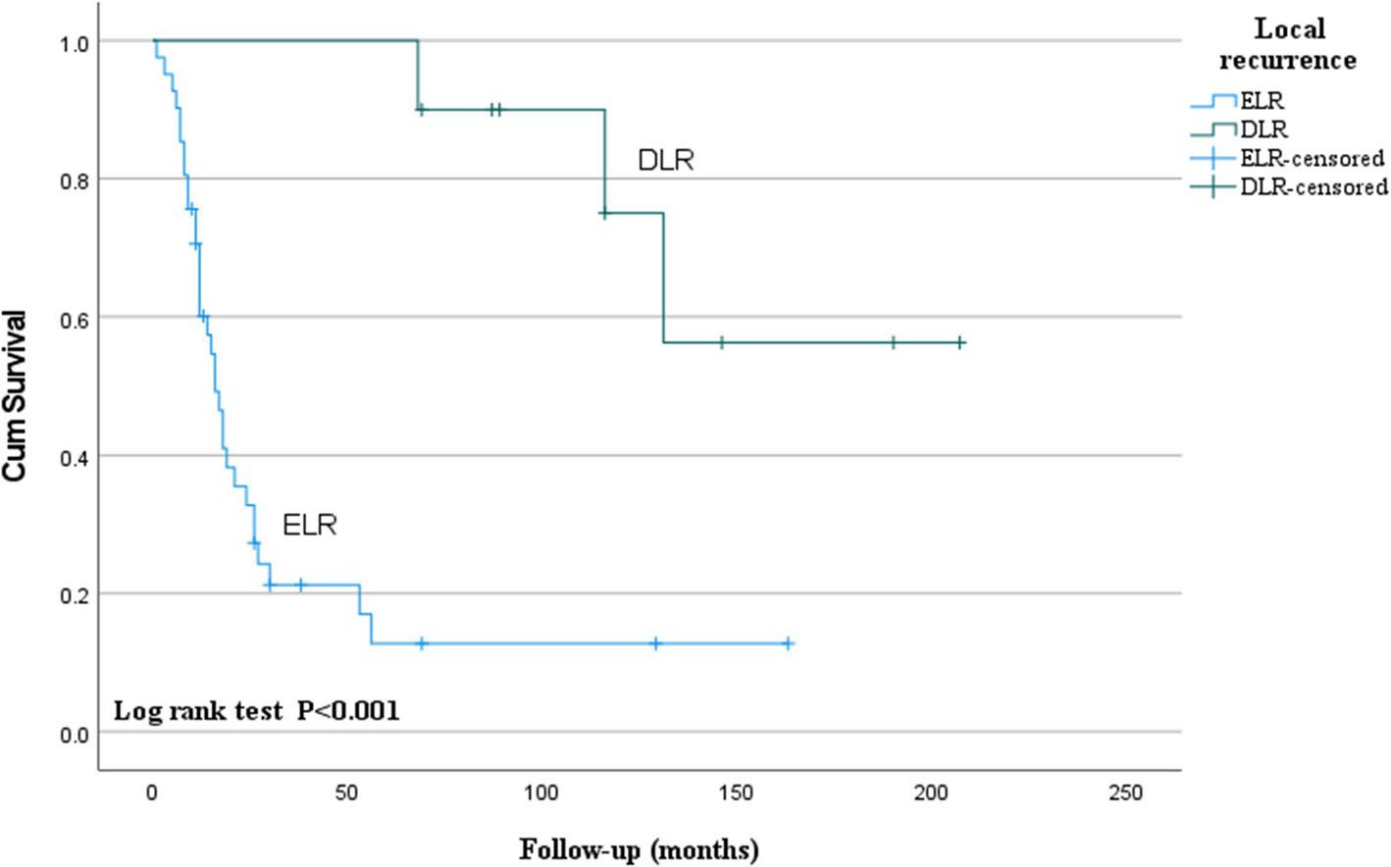
Kaplan–Meier survival analysis based on early local recurrence and delayed local recurrence

We conducted survival analysis using Kaplan–Meier analysis and the log-rank test to assess the survival outcomes of ELR and DLR (Fig. 2 and Table 6). The mean survival estimate for the overall local recurrence was 69 ± 12 months. For ELR, the mean survival estimate was 37 ± 9 months, while for DLR, it was 165 ± 19 months. The log-rank test results indicated a significant difference in cumulative survival rates between these two groups (*p* < 0.001).

**Table 6:** Means and medians for survival time of patients with local recurrence

Local recurrence	Mean^a^				Median			
	Estimate	Std. error	95% confidence interval		Estimate	Std. error	95% confidence interval	
			Lower bound	Upper bound			Lower bound	Upper bound
Early recurrence	36.8	8.8	19.6	54.0	16.0	2.0	12.12	19.9
Delayed recurrence	165.2	18.8	128.3	202.1				
Overall	68.9	12.1	45.2	92.6	21.0	4.3	12.5	29.5

^a^Estimation is limited to the largest survival time if it is censored

## Discussion

OSCC often experiences frequent recurrences. DLR is rarely observed in OSCC, and its exact cause is not well-established. In this study, we investigated the epidemiological and histopathological characteristics of patients with DLR and analyzed the correlations through statistical methods by following up with OSCC patients over a period of 17 years to understand the factors contributing to DLR occurrence.

In previous literature, local recurrence rates have varied from 5.44% to 29.04% [3–11]. In this study, the local recurrence rate was 25.89%, with an average time interval to tumor recurrence after surgery of 17 ± 23 months. Among the cases of local recurrence, 33.33% occurred within 6 months, 54.90% occurred within 12 months, and only 19.61% occurred after 24 months. Tumor recurrence occurs frequently within the first 6–12 months and is common within 2 years. Therefore, it is advisable to have close clinical monitoring at intervals of 4 weeks for at least the initial 6 months after surgery, and within the first year, follow-up visits every 2–3 months, along with imaging tests such as CT and MRI performed at intervals of 3–6 months within the first year. Neck ultrasound may be used to evaluate suspected lymph node metastasis. After 1-year post-surgery, positron emission tomography (PET)-CT demonstrated high sensitivity (92%) and specificity (91%) in detecting cancer recurrence in the head and neck region [12]. Clinical examination is the key to detecting delayed recurrences. Although there is limited evidence to support long-term imaging surveillance in patients with negative imaging results [13, 14], late recurrences can also occur. Therefore, regular and long-term follow-up observation including imaging surveillance may still be warranted.

There have been studies aimed at identifying variables related to local recurrence [10, 15–18], but research on factors associated with DLR is scarce and not well-documented. In this study, we examined the correlation between DLR and various variables, including gender, age, primary tumor site, T stage, N stage, histological grading, depth of invasion (DOI), lymphovascular invasion, perineural invasion, alcohol, smoking, RT, and ND. The results of the chi-square test indicated that there were no statistically significant differences in the epidemiological features between the ELR and DLR groups (*p* > 0.05). This suggests that there is no statistically significant difference in the distribution of the examined variables between the ELR and DLR groups. However, this does not mean that these variables have no impact on DLR. In this study, binary logistic regression was used to assess the influence of multiple variables on ELR and DLR. The logistic regression model did not identify any single factor that was significantly predictive of ELR or DLR. In contrast, ANOVA was used to compare differences in recurrence intervals based on various histopathological factors, revealing that histological grade significantly impacts the time to recurrence (*p* = 0.031). The combined use of these statistical methods provides a comprehensive understanding of how different factors correlate with recurrence patterns and intervals. There are previous studies suggesting that histological grading can influence recurrence [3, 11, 18]. One study suggested an association between histological grading and early tumor recurrence [3]. Another study indicated that histological grading can influence regional recurrence [19]. In summary, histological grading is shown to be an important factor in determining the timing of local recurrence, whether it is early or delayed. In other words, tumors with lower histological grading tend to have delayed recurrence.

While we have identified that histological grading influences the timing of local tumor recurrence, it is still challenging to predict the causes or likelihood of recurrence. The importance of the resection margin in relation to tumor recurrence has been well-established in several studies [20–22]. In this study, all patients had clear surgical margins ranging from 5 to 15 mm. However, even when the surgical margins are histologically tumor-free, local recurrences may still occur in 10–30% of patients [23]. Residual tumor and field cancerization can be helpful in explaining these phenomena and the cause of local recurrence [23–26]. Tumor cells that remain in the margins after surgery may eventually develop into a recurrence, but they are too small in number or size to be detected by routine histopathology. There are several studies suggesting that molecular diagnostic approaches are helpful in detecting such residual tumors. There are various detection methods, including TP53, hLy6D, and miRNA expression. The sensitive detection of residual cancer cells in the margins is crucial, and there is research suggesting that analyzing liquid biopsies can be helpful [23]. To treat these cells, postoperative radiotherapy or postoperative chemoradiotherapy can be applied. The concept of field cancerization was first introduced by Slaughter et al. in 1953 [27]. They observed that the entirety of the epithelium adjacent to the tumor displayed multiple distinct areas of malignancy. Numerous molecular techniques provided clear evidence in support of the concepts [28–30]. Various methods for the detection and visualization of field cancerization, including specific genomic markers, growth factors, vascular markers, dysplasia grading, loss of heterozygosity analysis, eIF4e, narrow band imaging, and brush biopsy, have been proposed, but they have not yet been standardized, and the entire process remains controversial [23, 24, 26]. This field cancerization treatment is more effective with systemic interventions rather than surgical excision and radiotherapy, and there is research indicating that 5-fluorouracil is highly effective [23, 31].

Although results may vary across different studies, many have analyzed the factors (i.e., tumor size, lymph node metastasis) that affect the survival rate of oral squamous cell carcinoma [18, 32–38]. Additionally, patients with recurrences have significantly lower survival rates compared to those without recurrences [11, 39, 40]. However, few studies have analyzed survival rates based on the timing of recurrence. In this study focusing on patients with local recurrence, Kaplan–Meier survival analysis revealed that the estimated survival period of patients with DLR was approximately 4.5 times longer than that of patients with ELR. The results of this study suggest that higher malignancy in cancer is associated with faster tumor recurrence and negatively impacts patient survival rates. This research concludes that among patients with recurrences, those with delayed recurrence have a better prognosis compared to those with early recurrence.

## Conclusions

Local recurrence is more common within the first 12–24 months following initial tumor resection surgery, while DLR after 2 years is rare. Predicting the recurrence interval is possible based on histological grading, with poorly differentiated histology associated with earlier recurrence. Consequently, patients with a higher histological grading may require close follow-up management during the initial 2 years. Even in cases of low cancer malignancy and no clinical symptoms, DLR can occur, emphasizing the need for long-term and periodic follow-up checks.

## Data Availability

Not applicable.

## Abbreviations

OSCC: Oral squamous cell carcinoma
DLR: Delayed local recurrence
ELR: Early local recurrence
DOI: Depth of invasion
RT: Radiation therapy
ND: Neck dissection
FOM: Floor of mouth
RMT: Retromolar trigone
BM: Buccal mucosa
PIOS: Primary intraosseous squamous cell carcinoma
CT: Computed tomography
MRI: Magnetic resonance imaging
PET: Positron emission tomography
ANOVA: Analysis of variance
TP53: Tumor protein 53
hLy6D: Human lymphocyte antigen 6 family member D
eIF4e: Eukaryotic translation initiation factor 4E
